# The Spatial Learning Task of Lhermitte and Signoret (1972): Normative Data in Adults Aged 18–45

**DOI:** 10.3389/fpsyg.2022.860982

**Published:** 2022-03-16

**Authors:** Alana Collins, Michael M. Saling, Sarah J. Wilson, Graeme D. Jackson, Chris Tailby

**Affiliations:** ^1^Florey Institute of Neuroscience and Mental Health, Parkville, VIC, Australia; ^2^Melbourne School of Psychological Sciences, The University of Melbourne, Parkville, VIC, Australia; ^3^Department of Neurology, Austin Health, Heidelberg, VIC, Australia; ^4^Department of Clinical Neuropsychology, Austin Health, Heidelberg, VIC, Australia

**Keywords:** memory, spatial learning, normative data, object-location learning, arbitrary associate learning, amnesia, temporal lobe epilepsy

## Abstract

**Objective:**

The Spatial Learning Task of Lhermitte and Signoret is an object-location arbitrary associative learning task. The task was originally developed to evaluate adults with severe amnesia. It is currently used in populations where the memory system either is not yet fully developed or where it has been compromised (e.g. epilepsy, traumatic brain injury, electroconvulsive therapy, cerebrovascular disease and dementia). Normative data have been published for paediatric cohorts and for older adults, however no data exist for the intervening adult years.

**Method:**

Here, we address this gap, collecting normative data from 101 adults aged 18–45.

**Results:**

Our data indicate that performance on the Spatial Learning Task is not influenced by age, gender, level of education or overall IQ. Less than 10% of the variance in learning scores is associated with variability in verbal memory. Ninety percent of participants achieved perfect scores on two successive trials (T2Cr) within five or fewer trials on the Spatial Learning Task. A T2Cr score of 6 is suggestive of impairment and a T2Cr score of 7 or more is statistically abnormal.

**Conclusion:**

These data expand the clinical utility of the Spatial Learning Task in the adult population. Future work should examine performance in lower IQ cohorts, including intellectual disability, and explore sensitivity to disease factors such as laterality of mesial temporal lobe damage.

## Introduction

The *Spatial Learning Task* of Lhermitte and Signoret (1972) is an object-location arbitrary associative learning task. Since its introduction, it has been used in a variety of clinical conditions to assess elementary levels of mnestic function. It has most commonly been employed in case studies of amnesia (Lhermitte and Beauvois, 1973; Ponsford and Donnan, 1980; Duyckaerts et al., 1985; Vighetto et al., 1991; Haslam et al., 1997), or in paediatric studies of the developing memory system (Anderson and Lajoie, 1996; Skuy et al., 2001; Anderson et al., 2001a; Pentland et al., 2003) and central nervous system insults during the developmental period (Northam et al., 1995; Anderson et al., 1997a,b, 2001b, 2004, 2005; Lindgren et al., 2004; Brunsdon et al., 2007; Aragón et al., 2008). The task differentiates severe (Lhermitte and Signoret, 1972), but not mild (Ponsford et al., 1980), mnestic disturbances.

In addition to its utility in individuals with low mnestic abilities (Walsh, 1985, 1994), the *Spatial Learning Task* has a number of other desirable features. It is simple to explain to patients and can be completed based solely on pointing responses, making it useful in a range of otherwise difficult to assess populations (non-English speaking, aphasic, learning disorders, visuoconstructional deficits, etc.). With nine stimulus elements, it is ‘supraspan’ for most individuals without being overwhelming. It also has a number of practical benefits including the use of simple, readily transportable stimulus materials and objective scoring.

The utility of any psychometric instrument is, however, limited by the availability of normative data. Norms for the *Spatial Learning Task* have been developed for paediatric cohorts (Anderson and Lajoie, 1996) and for older adults (Wardill et al., 2009); however, norms covering young to middle adulthood are not, to the authors’ knowledge, available. Such norms would be very useful given the range of clinical situations in which evaluation of rudimentary mnestic abilities is required in this age band (e.g. traumatic brain injury, hypoxic brain injury and neurosurgical evaluation).

The purpose of the present study is to obtain normative data on the *Spatial Learning Task* of Lhermitte and Signoret (1972) for adults aged 18–45. We also aim to evaluate the relationship between performance on the *Spatial Learning Task* and clinical/demographic variables such as age, overall intellectual ability, years of education, gender and other measures of mnestic function. We hypothesise that performance will be independent of these variables, given previous findings in older adults (Wardill et al., 2009) and the suggestion that this elementary mnestic task can be successfully performed *via* a range of cognitive strategies (e.g. verbal and/or nonverbal; Pentland et al., 2003).

## Materials and Methods

### Participants

We recruited 101 participants aged between 18 and 45 years. Mean age was 25.3 years (SD = 6.8); 46% of participants were male (Table 1). Mean number of years of education was 15.3 years (SD = 2.8) and mean level of intellect, derived from the WASI-II FSIQ-2, was 115.5 (SD *=* 8.45).

**Table 1:** Demographic characteristics of the sample (*N* = 101).

	Age (M; F)^*^	Education (M; F)	IQ (M; F)
*Mean*	25.3 (25.4; 25.2)	15.3 (15.0; 15.6)	115.5 (116.5; 114.7)
*SD*	6.8 (6.9; 6.7)	2.8 (2.3; 3.1)	8.5 (8.2; 8.7)
*Range*	18–45 (18–45; 18–41)	12–24 (12–20; 12–24)	86–130 (86–130; 94–129)

*Education = Years of education; IQ = WASI-II FSIQ-2 score;*

* *each column reports sample means, SDs and Ranges for the overall sample, with male (M) and female (F) specific values reported in parentheses.*

Fifty of the participants were undergraduates, 51 were members of the community. Participants were unpaid volunteers recruited by means of posters and convenience sampling. The study had approval from the relevant Human Research Ethics Committees and written informed consent was obtained from all participants.

Participants were screened on a self-report medical screening questionnaire at the beginning of the study. Participants who reported a history of neurological injury, psychiatric illness, severe head injury, stroke, seizures or other serious medical conditions were excluded from participation. All participants spoke English fluently; 80% had English as their first language.

### Materials

**Figure 1:**
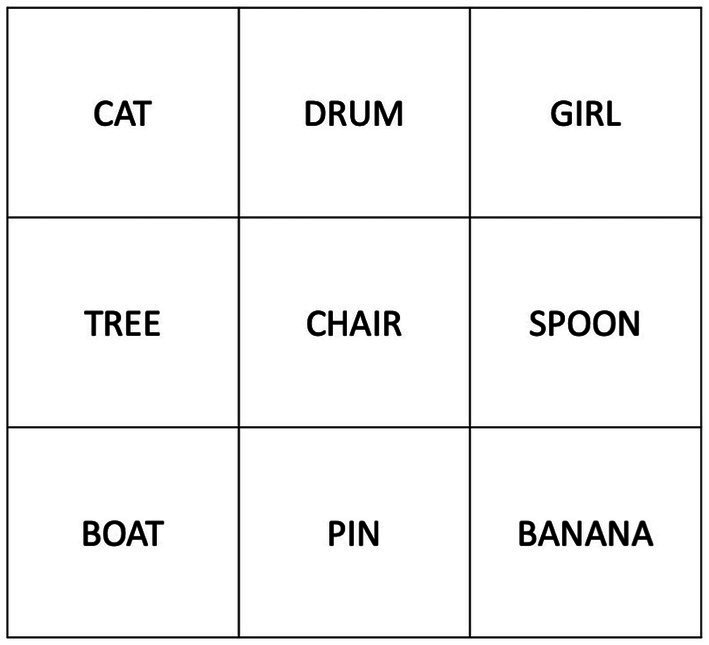
Stimulus arrangement in the Spatial Learning Task of Lhermitte and Signoret (1972). Photographs were used in the original study, with simple line drawings used in a number of subsequent studies (see Anderson and Lajoie, 1996 for an example) including the present study.

The *Spatial Learning Task* (Lhermitte and Signoret, 1972) assesses the ability to learn an arbitrary relationship between objects and their associated locations in immediate personal space. There are nine stimulus cards (line drawings of everyday objects), each occupying a particular position within a 3 × 3 grid (Figure 1). The stimulus cards are presented in a pre-specified random order, one at a time, with participants instructed to remember their spatial locations. After all, nine picture-location associations have been demonstrated once each picture card is then presented individually and the participant asked to identify its corresponding grid location. The examiner either confirms a correct response or demonstrates the correct location by placing the card in the correct grid position. Testing is continued until all cards have been placed correctly on two consecutive trials, or until 10 trials have been administered, whichever occurs first (Wardill et al., 2009). After ~30 min of intervening, unrelated activity following completion of the last learning trial, delayed recall is assessed by presenting each individual card and asking the participant to indicate its position of the grid. Derived scores include:

- *Trials to Criterion (T2Cr)* recorded as the second of two successive error free trials (i.e. for a participant scoring 9/9 and 9/9 on trials 1 and 2, *T2Cr* = 2; for a participant scoring 9/9, 8/9, 9/9 and 9/9 on trials 1, 2, 3 and 4, *T2Cr* = 4). *T2Cr* was the primary variable of interest. This corresponds to the *trials to criterion* employed by Wardill et al. (2009).
- *Trials to first perfect score (TFP)* recorded as the first error free trial (i.e. for a participant scoring 9/9 and 9/9 on trials 1 and 2, *TFP* = 1; for a participant scoring 9/9, 8/9, 9/9 and 9/9 on trials 1, 2, 3 and 4, *TFP* = 1). *TFP* corresponds to *SLCRIT* as reported in Anderson and Lajoie (1996).
- *Total score,* total correct responses over 10 trials (extrapolated for those who attain *T2CR* earlier).
- *Delayed recall*, number of correct responses after a 30-min delay.

#### Rey-Osterrieth Complex Figure Test

This task was used as a measure of visuoconstructive skills and visual memory (Corwin and Bylsma, 1993). Participants are asked to copy a figure and draw it again from memory immediately and after a 30-min delay. Scores for copy, immediate recall, delayed recall and recognition were recorded.

#### The Rey Auditory Learning Test

This task was used as a measure of verbal memory and learning. List A of the Rey Auditory Learning Test (RAVLT; Lezak et al., 2012) was used. The list contains 15 words that were read out loud to participants at a rate of one word per second, always in the same order. The total number of words recalled across the five learning trials, on the interference trial, on the post- interference trial and on the 30-min delayed recall trial were recorded.

#### Wechsler Abbreviated Scale of Intelligence Second Edition

The Wechsler Abbreviated Scale of Intelligence Second Edition (WASI-II) provides a brief estimate of general intellectual functioning (Wechsler, 2011). The 2-item version (Matrix Reasoning; Vocabulary) was administered and scored according to standardised procedures. Performance on each subtest was converted to an age-adjusted score for each item and then a prorated intelligence score (FSIQ-2), according to the scoring manual.

### Procedure

Assessments were conducted in person, in a quiet room, either in a testing room on campus or at the participant’s home. Cognitive tasks were administered in the following order: Spatial Learning Task, Rey-Osterrieth Complex Figure Test (RCFT), RAVLT and the WASI. Delayed recall conditions were then performed in the same order. All assessments were administered in a single 60-min session.

### Data Analysis

All data were analysed using Rstudio, with *p* < 0.05 (two tailed) used to determine statistical significance.

Prior to analysis, all variables were examined for accuracy of data entry, missing values and assumptions for parametric analysis. Data were initially inspected using box plots and scatter plots and then investigated for normality using Shapiro-Wilk test. Data were also investigated using median absolute distance (cut-off value of 3) and chi-squared analysis of Mahalanobis distance for univariate and multivariate outliers. Univariate outliers identified were reviewed and included in all analyses. No multivariate outliers were identified. *Trials to Criterion (T2Cr)* from the *Spatial Learning Task* was used in all correlation and regression analyses as it contained the least outliers and was the least skewed Lhermitte variable.

Normative datasets frequently take into account demographic factors such as age, gender and level of education. We used non-parametric tests including Spearman rank correlations to assess the relationship between *Spatial Learning Task* performance and demographic variables (age, gender, years of education and intellect). Non-parametric tests were used as *Trials to criterion (T2Cr)* was positively skewed and normality could not be produced by any transformation. To further explore possible relationships between demographic variables and *Spatial Learning Task* performance a series of regression analyses were used. Based on visual inspection and comparison of model fit for both linear and quadratic solutions, quadratic terms for age, years of education and prorated intelligence were used. To reduce collinearity between linear and quadratic terms, centred scores for demographic variables were used in significance tests. Stepwise hierarchical linear regression with linear and quadratic terms for age, gender, intellect and years of education as predictors, including all possible interactions, were conducted.

For descriptive purposes, we present normative data for the *Spatial Learning Task* variables: *Trials to criterion*, *Trials to First Perfect Score, Total Score* and *Delayed Recall Score*. Cut-scores for *Trials to criterion* and *Trials to First Perfect Score* were also calculated, indicating the percentage of people scoring at better than a given score. We also report empirical cumulative density functions for scores on individual trials as well as total (cumulative) scores from Trial 1 through to Trial 5 (e.g. sum[T1], sum[T1,T2], sum[T1,T2,T3], sum[T1,T2,T3,T4] and sum[T1,T2,T3,T4,T5]).

To investigate the possible relationship between *Spatial Learning Task* scores and other cognitive constructs, we calculated Spearman rank correlations with scores on the *RAVLT*, *RCFT* and *WASI-II* subtests (Vocabulary and Matrix Reasoning).

We used between groups t-tests to compare *T2Cr* and *TPF* in our sample with the corresponding summary statistics reported in a paediatric sample (Anderson and Lajoie, 1996) and in a sample of older adults (Wardill et al., 2009).

## Results

### *Spatial Learning Task* Performance

#### Most Healthy Adults Achieve Perfect Scores Within the First Few Trials

**Figure 2:**
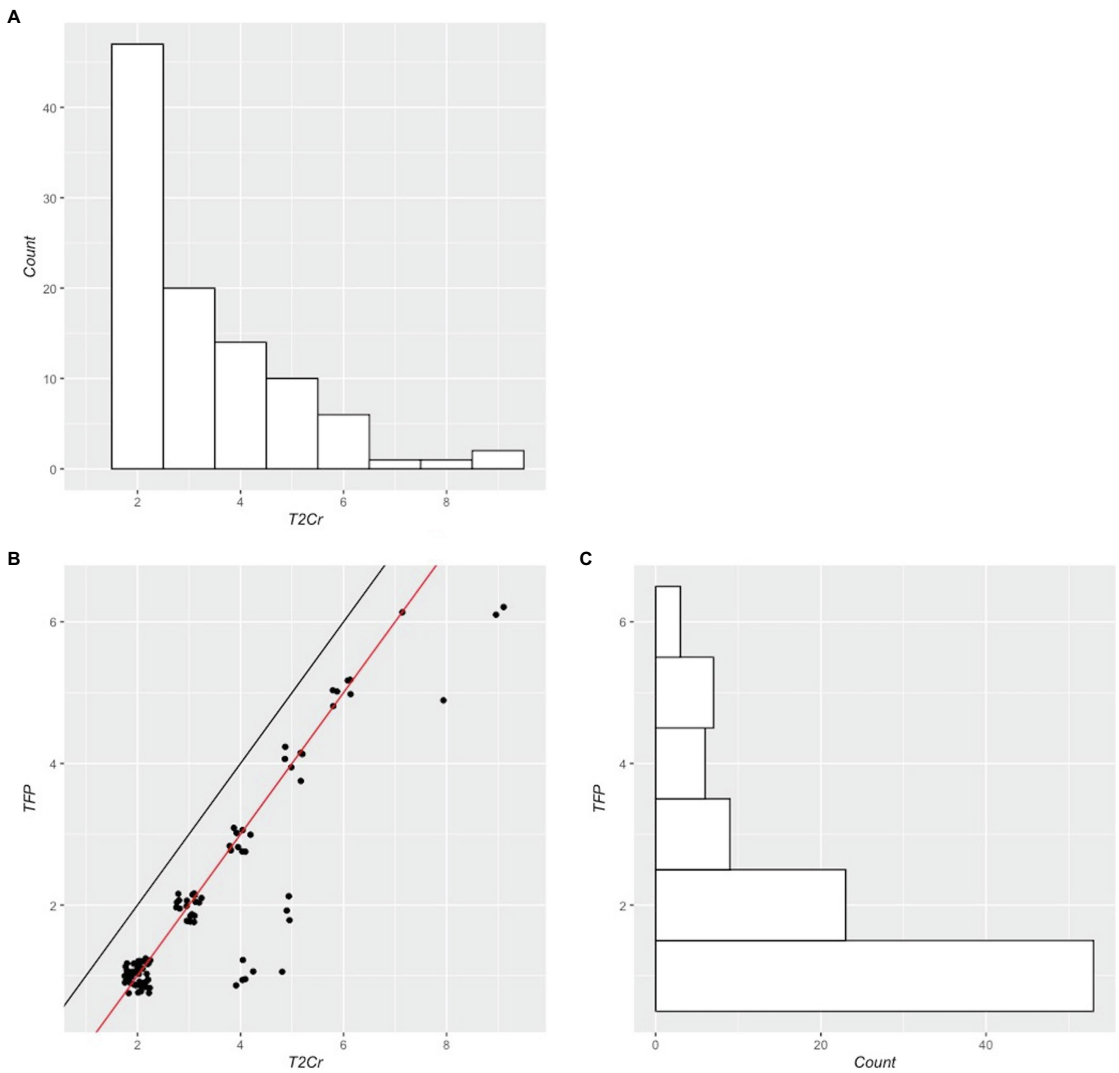
Scatterplot **(B)** and marginal histograms of T2Cr **(A)** and TFP **(C)**. In **B**, the black diagonal line shows the unity line, and the red diagonal line shows the best possible T2Cr score for a given TFP score (i.e. when the first trial on which a perfect score is obtained is followed by another perfect score on the immediately succeeding trial). Data points below the red diagonal indicate that one or more trials contained errors after TFP was attained, suggesting unstable learning. Data points have been jittered in x and y to improve visualisation.

The distribution of scores for *Trials to Criterion (T2Cr),* the primary measure of interest, and *Trials to First Perfect Score (TFP)* are presented in Figure 2, along with their scatterplot. Scores for *T2Cr* and *TFP* were both positively skewed (skew = 1.47 and 1.36, respectively), deviating significantly from normality (Shapiro-Wilk tests, *p* < 0.001). A ceiling effect is readily apparent in our sample of controls, with most participants attaining *TFP* and *T2Cr* within the first few trials. For the majority of participants, their first perfect score is followed by a subsequent perfect score (thereby attaining *T2Cr*); only 12 of 101 participants (11%) returned an imperfect score immediately following their first perfect score trial (*TFP*), before eventually obtaining two successive perfect scores (*T2Cr*; see Figure 2B).

#### Spatial Learning Task Performance Is Not Related to Age, Years of Education, Gender or IQ

**Figure 3:**
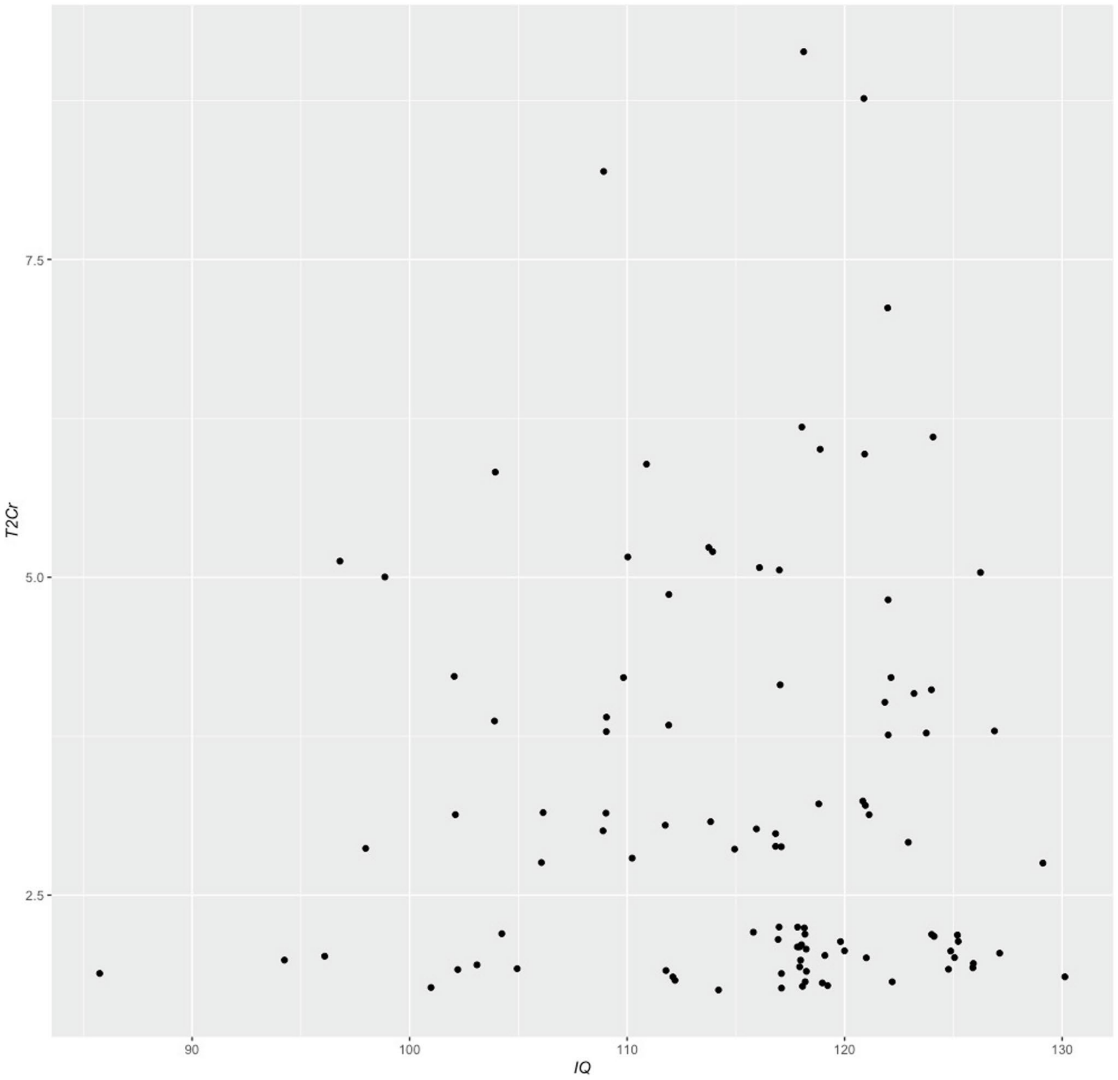
Relationship between Spatial Learning Task scores (T2Cr) and intellect (IQ). Data points have been jittered in x and y to enable visualisation of overlapping data points improve visualisation.

Before presenting normative data, we first checked for significant relationships between *T2Cr* and demographic variables, to determine whether stratification on these variables would be required. Spearman bivariate correlation analyses did not identify significant correlations between *T2Cr* and age, years of education, gender or IQ (see Table 2 and Figure 3).

**Table 2:** Bivariate correlation analysis (Spearman’s rho) between Trials to Criterion and Age, Years of Education, Gender and IQ.

	*T2Cr*	
	*r*	*p*
Age	0.136	0.18
Years of Education	0.070	0.49
Gender	−0.103	0.31
IQ	−0.096	0.34

*T2Cr = Spatial Learning Task Trials to Criterion; IQ = prorated intellect, FSIQ-2.*

We also used separate multiple regression models that included quadratic terms to examine for significant nonlinear relationships between *Spatial Learning Task* performance (*T2Cr*) and age, IQ and years of education. None of these variables significantly predicted *Spatial Learning Task* performance (*T2Cr*; in all instances *p* > 0.29 for the F test on the overall model, and *p* > 0.11 for individual demographic predictors). Consequently, in the following section, we present normative data collapsed across age, gender, IQ and years of education.

### Normative Data for the Spatial Learning Task

The percentage of the sample having attained *TFP* and *T2Cr* at each *Spatial Learning Task* trial are presented in Table 3. Ninety-one of 101 participants (90.1%) obtained at least one perfect score (*TFP*) within four or fewer trials, with more than half of the sample (53 of 101, 52.4%) obtaining a perfect score on the first trial. Forty-seven of 101 participants (46.5%) reached *T2Cr* from the second trial (the best possible performance for *T2Cr*), while 91 (90.1%) reached *T2Cr* after five trials or less. The highest observed *T2Cr* score was nine trials, observed in one participant.

**Table 3:** Cumulative number of participants attaining T2Cr and TFP on the Spatial Learning Task as a function of trial number.

*Trial Number*	Cumulative number of individuals (%) attaining *T2Cr*	Cumulative number of individuals (%) attaining *TFP*
1	*NA*	*n* = 53 of 101(52.5%)
2	*n* = 47 of 101(46.5%)	*n* = 76 of 101(75.2%)
3	*n* = 67 of 101(66.3%)	*n* = 85 of 101(84.1%)
4	*n* = 81 of 101(80.2%)	*n* = 91 of 101(90.1%)
5	*n* = 91 of 101(90.1%)	*n* = 98 of 101(97.0%)
6	*n* = 97 of 101(96.0%)	*n* = 101 of 101(100.0%)
7	*n* = 98 of 101(97.0%)	*–*
8	*n* = 99 of 101(98.0%)	*–*
9	*n* = 101 of 101(100.0%)	*–*

Participants made very few mistakes while learning the object-location pairings, with a total of 47 participants (46.5%) attaining the maximum possible *Total Score* of 90. Only four participants (4%) obtained a total score ≤ 81. All but one participant performed at ceiling (9 out of 9) in the *Delayed recall* condition.

Thus, based on our sample, a *T2Cr* score of 6 is suggestive of impairment (only 9.9% of the control sample have a *T2Cr* score of 6 or greater), and a *Trials to Criterion* score ≥ 7 indicates statistically abnormal performance (only observed in 4% of our control sample). A total score ≤ 81 is statistically abnormal (only observed in 4% of our control sample), and a delayed recall score below 9 is also statistically abnormal (in a participant who has attained criterion across the learning trials, as was the case for all of our participants).

**Figure 4:**
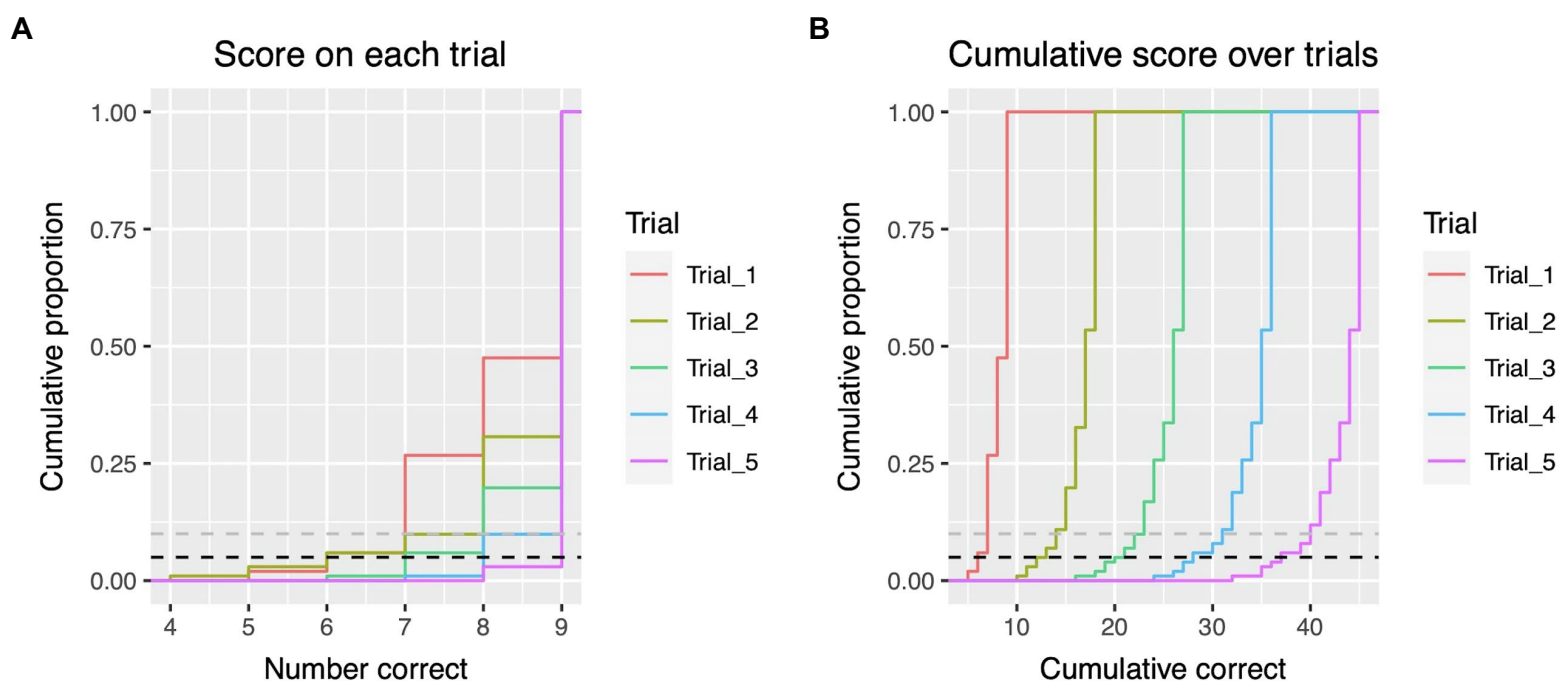
Empirical cumulative density functions for total scores on individual trials **(A)** and summed across trials **(B)**. Black dashed line shows 5th percentile; grey dashed line shows 10th percentile.

We also calculated empirical cumulative density functions (ECDFs) for scores on individual trials and for cumulative scores calculated across increasing numbers of trials (Figure 4). Scores falling below the 10th and 5th percentile for individual trial scores and for cumulative scores are shown in Table 4.

**Table 4:** Cut-off scores for individual trials, and for cumulative scores across trials, at which performance on the Lhermitte board falls below the 10th and 5th percentiles.

		Trial 1	Trial 2	Trial 3	Trial 4	Trial 5
Individual Trial Score	<10th percentile	<7	<8	<8	<9	<9
	<5th percentile	<6	<6	<7	<8	<9
Cumulative score	<10th percentile	<7	<14	<23	<31	<40
	<5th percentile	<6	<13	<21	<28	<37

### Spatial Learning Task Performance Is Moderately Associated With Verbal Learning Ability

Spearman bivariate correlation analysis (Table 5) revealed weak but significant negative correlations of *T2Cr* with *RAVLT Total* score ( ρ = −0.270, *p* = 0.006, *r^2^* = 0.07) and *RAVLT Delay* score ( ρ = −0.234, *p* = 0.019, *r^2^* = 0.05), indicating that learning and later recalling more words on the RAVLT is related to better performance on the *Spatial Learning Task*. There was no significant correlation between *T2Cr* and scores on the RCFT, or scores on the WASI-II subtests. As already noted, *T2Cr* was not associated with overall IQ (Table 2).

**Table 5:** Bivariate correlation analysis (Spearman’s rho) between T2Cr and RCFT (immediate delay), RAVLT (total and delay), WASI Vocabulary and WASI Matrix Reasoning.

	*T2Cr*	
	ρ	Value of *p*
RCFT Immediate Delay	−0.137	0.172
RCFT Long Delay	−0.149	0.147
RAVLT Total	−0.270	0.006
RAVLT Delay	−0.234	0.019
WASI Vocabulary	−0.114	0.258
WASI Matrix Reasoning	−0.031	0.758

*RCFT Immediate delay, Rey-Osterrieth Complex Figure Task, Immediate delay; RAVLT Total, Rey’s Auditory Verbal Learning Test, Total Score; RAVLT Delay, Rey’s Auditory Verbal Learning Test, Delay Score; and WASI, Wechsler Abbreviated Scale of Intelligence Second Edition*.

### Comparison With Published Normative Data From Older Adult and Paediatric Cohorts

The normative obtained from our sample (Tables 3, 4) appears comparable to that reported in the older adult sample (age 65+) provided by Wardill et al. (2009). We only had access to summary statistics for this older adult cohort, so we compared samples using parametric methods. A between groups t-test, based on the mean and standard deviation of *T2Cr* observed herein and those reported in Wardill et al. (2009), was not significant (mean diff = −0.31, *t* = −1.53, *p* = 0.13, *Cohen’s D* = 0.19).

Anderson and Lajoie (1996), in their paediatric normative study, reported means and standard deviations for *TFP* rather than *T2Cr*. Comparison with the oldest paediatric age band reported in Anderson and Lajoie (1996)—age 13—indicated that performance was significantly better in our adult sample (mean diff = −0.49, *t* = −2.34, *p* = 0.02, *Cohen’s D* = 0.39).

## Discussion

The purpose of this study was to collect adult normative data on the *Spatial Learning Task* of Lhermitte and Signoret (1972). Ninety percent of participants achieved perfect scores on two successive trials (*T2Cr*) within five or fewer trials. Our data indicate that a *T2Cr* score of 6 is suggestive of impairment and a *T2Cr* score of 7 or more is statistically abnormal. At a practical level, we observed that roughly 10% of participants obtain an imperfect score after their first perfect score. Thus, we recommend continuing administration of the task until two successive perfect scores have been obtained, thereby demonstrating stable memory for the object-location associations.

The lack of dependence of *Spatial Learning Task* performance on age, gender, level of education or IQ is a desirable property that simplifies test interpretation and widens the utility of the instrument. Typically, when interpreting a particular test score obtained from a given individual these demographic factors must be accounted for, often through stratified normative data or prediction equations. Our data imply this is not the case for the *Spatial Learning Task*, at least across the demographic range of our cohort.

Previous research has highlighted the importance of individual differences (Waller, 2000; Lopez et al., 2020), especially gender differences (Silverman and Eals, 1992), in measures of object-location learning. For instance, the meta-analysis of Voyer et al. (2007) reported an overall effect favouring women on Object-Location Memory tasks. This gender effect was age dependent, emerging over the age of 13, and was greater for *recall* (versus *recognition*) and for *categorical* (e.g. top/bottom, left/right; versus *coordinate*, e.g. distance displacement error) formats, though was not apparent in all studies (see ‘Discussion’ in Rahman et al., 2011). Our own results using the *Spatial Learning Task*, which can be considered a *categorical recall* task, did not reveal a gender effect. This could reflect the simplicity of the *Spatial Learning Task* itself (i.e. ceiling effects in the normal population precluding the presence of a gender effect) and its multidetermined nature (i.e. that it is not a ‘pure’ spatial learning task, as considered further below).

The ceiling effect that we observed, with the majority of participants attaining perfect performance across the first few trials, is expected of measures that sample the low end of functional ability (Kaplan and Saccuzzo, 2017), as the *Spatial Learning Task* does for memory (Walsh, 1985). In clinical practice, the assessment of such basic mnestic abilities is often carried out informally. Typical examples include asking patients to recall a short list of unrelated words or to learn the identity and location of a small number of objects hidden around the room by the examiner, with ‘rules of thumb’ guiding the interpretation of performance (Strub and Black, 2000; Lezak et al., 2012). The *Spatial Learning Task* affords the opportunity to evaluate such basic mnestic abilities quantitatively, and the normative data reported here extend the utility of this measure to the adult population.

It is important to note that while poor performance on the *Spatial Learning Task* provides evidence of significant mnestic compromise, normal performance cannot be taken to reflect unimpaired mnestic function. The authors’ clinical experience is that individuals can exhibit subtle to moderate declines on measures such as the Rey Auditory Verbal Learning Test without showing any compromise on the *Spatial Learning Test*. Thus, the *Spatial Learning Test* should not be used or interpreted in isolation, but rather considered alongside other measures of memory ability. A dense amnestic picture would, by definition, be expected to show poor performance across multiple measures of anterograde memory.

Among the other cognitive tasks that we administered, the only significant relationship with *T2Cr* that we observed was with the RAVLT (*total words recalled across learning trials* and *delayed recall*). This relationship was weak, only accounting for between 5 and 7% of variance in Spatial Learning Task scores. This suggests, as has been posited previously (Pentland et al., 2003), that while the task is notionally a visuospatial associative learning task, verbal learning and memory can contribute to task performance. The use of simple, familiar objects in conjunction with an orderly grid arrangement of locations makes the task performable *via* a range of strategies, from purely verbal (e.g. by approaching the task as one of verbal associate learning: ‘Chair – Middle’, ‘Kitten – Top Left’ and ‘Banana – Southeast’) to purely nonverbal and varying combinations thereof (see also Helmstaedter et al., 1995). This again points to the importance of considering performance on the *Spatial Learning Task* relative to other measures of memory ability.

A range of ‘purer’ measures of spatial memory have been developed, many with normative data available, such as the Brief Visuospatial Memory Test—Revised (Benedict et al., 1996); the Brown Location Test (Brown et al., 2007); the Location Learning Test (Bucks and Willison, 1997; Bucks et al., 2000); the modified Location Learning Test (Kessels et al., 2004, 2006); the Spatial Array Memory Test (Meador et al., 1990); the Spatial Location Test (Sanchez et al., 1997); the Visual Spatial Learning Test (Malec et al., 1992); and the 7/24 Spatial Recall Test (Gontkovsky et al., 2004; see also references cited in discussion of spatial memory and hemispheric lateralisation below). Such measures however are best suited to addressing the question of differences relative to typical performance in the normal population rather than the assessment of elementary levels of mnestic function, the principal question of interest in the present study.

When considered next to other published norms, our data suggest that performance on the *Spatial Learning Task* improves between age 13 and the youngest individuals in our adult cohort. Inspection of the normative data reported in Anderson and Lajoie (1996; e.g. Figure 3 therein) suggests that even in paediatric cohorts performance approaches ceiling, and hence that the improvement in the adult population is modest. This improvement likely reflects maturation of executive and metacognitive abilities brought to bear on the task over this developmental period (Anderson and Lajoie, 1996). For instance, Huizinga et al. (2006), using a latent variable analysis applied to a battery of executive tasks administered to a range of age groups, have shown that elements of executive function continue to develop throughout adolescence. There is also a large body of literature showing that the availability and effective deployment of memory strategies increases throughout childhood (Schneider and Bjorklund, 2002). Such metacognitive memory strategies emerge relatively early in childhood and continue to develop into early adolescence and beyond (Bjorklund et al., 2008; Roebers, 2014).

The normative values derived from our data are comparable to the older adult data of Wardill et al. (2009), implying that once adult performance levels on the task are attained they remain stable throughout adulthood. Our data are also consistent with the observations of Walsh, who stated that ‘Subjects of normal intelligence will almost invariably reach a perfect score in six trials or less’ (Walsh, 1985, p. 240). Ninety percent of participants in our cohort had returned at least one perfect score within four trials or less, and by the 6th trial, all 101 participants had returned at least one perfect score (Table 3).

Combing our own observations with those of others (Walsh, 1985; Wardill et al., 2009) suggests that poor performance in an adult can be considered a reliable marker of memory dysfunction that can be interpreted free of confounds from other demographic variables such as age, level of education and IQ. Further research targeting specific clinical populations is required to expand the clinical utility of the task. For instance, does the lack of dependence on IQ extend beyond the lower end of the IQ range sampled here to include those with mild to moderate ID? It would also be useful to relate performance on the task to real world metrics, to explore the ecological validity of the task for day-to-day memory functions (e.g. ratings from carers; prognostic value with respect to skill acquisition in clinical cohorts with severe memory impairment).

Further research is also required to determine whether unilateral temporal lobe compromise is sufficient to disrupt task performance, or whether bitemporal dysfunction is required. In our clinical experience, patients with evidence of bilateral mesial temporal dysfunction perform poorly on the *Spatial Learning Task*. This includes individuals with degenerative disease, such as Alzheimer’s disease, and also focal epilepsy patients with evidence of bitemporal disease (e.g. bilateral hippocampal sclerosis). However, the question remains, can performance be affected in the presence of unilateral disease/pathology. Considerable debate surrounds the question of hemispheric asymmetries and spatial learning (Saling, 2009). Clinically, we have seen individual cases of unilateral, right temporal lobe epilepsy who are impaired on the *Spatial Learning Task*, and the weight of published evidence does appear to suggest at least a right hemisphere bias for object-location memory more broadly (Smith and Milner, 1981, 1989; Abrahams et al., 1997; Baxendale et al., 1998; Bohbot et al., 1998; Nunn et al., 1998, 1999; Incisa della Rocchetta et al., 2004; Kessels et al., 2004; Stepankova et al., 2004; Crane and Milner, 2005; Glikmann-Johnston et al., 2008; Frisch and Helmstaedter, 2014; see also the meta-analysis by Kessels et al., 2001). A systematically collected, prospective case series would be required to address the question of potential lateralising significance (uni- versus bilateral; left versus right) of the *Spatial Learning Task.*

A limitation of the present study was reliance upon self-report of neurological and psychiatric history. Self-report, while time and resource efficient, is not as sensitive and specific as ‘gold standard’ approaches such as structured clinical interview, medical records review or re-examination (e.g. mood: (Coyne et al., 2001; Stuart et al., 2014); stroke: (Engstad et al., 2000; Woodfield et al., 2015); and hospitalisation: (Bergmann et al., 1998)). Additionally, the participants in this study comprise a sample of convenience rather than being actively matched to population demographics, and as a group are relatively high functioning (mean level of education = 15.3 years; mean IQ =115.5).

## Conclusion

We have obtained normative data for the *Spatial Learning Task* (Lhermitte and Signoret, 1972) from a sample of 101 participants aged 18–45. We have shown that performance is not influenced by age, gender, the number of years of education or overall IQ, and less than 10% of the variance in learning scores is associated with variability in verbal memory. Our data provide the basis for normative comparisons in adults: 90% of participants achieved perfect scores on two successive trials (*T2Cr*) within five or fewer trials; a *T2Cr* score of 6 is suggestive of impairment and a *T2Cr* score ≥ 7 is statistically abnormal. These data expand the clinical utility of the *Spatial Learning Task* in the adult population.

## Data Availability Statement

The raw data supporting the conclusions of this article will be made available by the authors, without undue reservation.

## Ethics Statement

The studies involving human participants were reviewed and approved by the University of Melbourne Human Research Ethics Committee. The patients/participants provided their written informed consent to participate in this study.

## Author Contributions

CT, MS, and SW planned and designed the study and coordinated recruitment and data collection. AC and CT analysed the data and drafted the manuscript. All authors contributed to the article and approved the submitted version.

## Funding

The Florey Institute of Neuroscience and Mental Health acknowledges the strong support from the Victorian Government and in particular the funding from the Operational Infrastructure Support Grant.

## Conflict of Interest

The authors declare that the research was conducted in the absence of any commercial or financial relationships that could be construed as a potential conflict of interest.

## Publisher’s Note

All claims expressed in this article are solely those of the authors and do not necessarily represent those of their affiliated organizations, or those of the publisher, the editors and the reviewers. Any product that may be evaluated in this article, or claim that may be made by its manufacturer, is not guaranteed or endorsed by the publisher.
